# Correction: The structure of the Ctf19c/CCAN from budding yeast

**DOI:** 10.7554/eLife.56553

**Published:** 2020-03-05

**Authors:** Stephen M Hinshaw, Stephen C Harrison

Hinshaw SM, Harrison SC. 2019. Rapid The structure of the Ctf19c/CCAN from budding yeast. *eLife* **8**:e44239. doi: 10.7554/eLife.44239.

Published 14, Feburary 2019

Although we had previously identified them, we accidentally misidentified amino acid substitutions in the pTDAO plasmid used for production of recombinant Ame1-Okp1. Database searches confirmed the Ame1 gene was likely originally amplified from *S. cerevisiae* strain YJM1355. The differences are not in residues explicitly modeled in our structure and do not affect the reported findings.

Original text:

We found that the Ame1-6His; Okp1 expression plasmid used in this study codes for an Ame1 amino acid point mutation in the disordered N-terminal extension not seen in our density map (L19P) and also lacks the codon for the final amino acid residue of Okp1 (H406).

Corrected text:

The Ame1-6His; Okp1 expression plasmid used in this study codes for Ame1 from *S. cerevisiae* strain YJM1355, which differs from S288c as follows: L97P and G269E. Neither residue is explicitly modeled in the deposited structure. The plasmid also lacks the codon for the final amino acid residue of Okp1 (H406).

The article has been corrected accordingly.

