# Supplementary material for: The structure of the Ctf19c/CCAN from budding yeast
Source: eLife. 2019 Feb 14;8:e44239. doi: 10.7554/eLife.44239 (PMC6407923; doi:10.7554/eLife.44239)
Supplement: Supplementary file 1. — Tables describing cryo-EM data collection and molecular modeling. [file elife-44239-supp1.docx]

**Supplementary file 1 – Data and model statistics**

| **Data collection and processing** | **Dataset 1** | **Dataset 2** | **Dataset 3** |
| --- | --- | --- | --- |
| Electron microscope | Polara | Polara | Polara |
| Voltage (kV) | 300 | 300 | 300 |
| Electron dose (e-/Å2) | 47 | 47 | 47 |
| Frames per movie | 40 | 40 | 40 |
| Time per frame (s) | 0.2 | 0.2 | 0.2 |
| Physical pixel (Å) | 1.23 | 1.23 | 1.23 |
| Number of movies | 2594 | 2948 | 9951 |
| Number of particles | 564366 | 699208 | 2551127 |
|  |  |  |  |
|  |  |  |  |
| **Model and map statistics** | **Half particle (refinement)** | **Full particle (C2)** | **Ctf3c-Iml3** |
| Particles for final map | 119469 | 265880 | 119469 |
| Resolution (Å) | 4.25 | 4.73 | 4.3 |
| Map B-factor for sharpening (Å2) | -189.1 | -259.3 | -182.8 |
|  | **Model properties** |  |  |
| Number of unique chains | 14 |  |  |
| Number of protein residues | 2372 |  |  |
| Number of atoms | 16332 |  |  |
|  | **Geometric parameters (RMSD)** |  |  |
| Bond length (Å) | 0.010 |  |  |
| Bond angle (degrees) | 1.375 |  |  |
|  | **Ramachandran statistics** |  |  |
| Residues favoured (%) | 85.45 |  |  |
| Residues allowed (%) | 14.42 |  |  |
| Residues disallowed (%) | 0.13 |  |  |
| Rotamer outliers (%) | 4.15 |  |  |
| Molprobity score | 2.66 |  |  |
| EMRinger score | 1.52 |  |  |
