# Supplementary material for: The structure of the Ctf19c/CCAN from budding yeast
Source: eLife. 2019 Feb 14;8:e44239. doi: 10.7554/eLife.44239 (PMC6407923; doi:10.7554/eLife.44239)
Supplement: Supplementary file 2. — Table describing the Ctf19c model. Each subunit is listed along with the number and identities of amino acid residues modeled. [file elife-44239-supp2.docx]

**Supplementary file 2 – Summary of the refined model**

| **Subcomplex** | **Protein** | **Total residues** | **Chain ID** | **Template for modelling** | **Procedure** | **Modelled residues** | **Poly-alanine** |
| --- | --- | --- | --- | --- | --- | --- | --- |
| COMA-Nkp1/2 | Nkp1 | 238 | G |  | Build de novo | 4-36; 40-82; 84-122; 135-177; 187-204 | 4-36; 135-177; 187-204 |
|  | Nkp2 | 153 | J |  | Build de novo | 2-80; 84-153 | 84-153 |
|  | Ame1 | 324 | I |  | Build de novo | 124-150; 170-276; 289-321 | 124-150; 266-276 |
|  | Okp1 | 406 | F | PDB 5MU3 | Build de novo; short peptide from PDB 5MU3 (K. lactis) | 162-224; 233-297; 323-386 | 162-224; 292-297 |
|  | Ctf19 | 369 | D | PDB 5MU3 | Dock and reassign sequence; substantial adjustment | 125-173; 177-201; 209-272; 279-284; 297-317; 327-368 | 125-128 |
|  | Mcm21 | 368 | C | PDB 5MU3 | Dock and reassign sequence; substantial adjustment | 153-187; 193-328; 337-353 |  |
| Chl4-Iml3 | Chl4 | 458 | E | PDB 4JE3; PDB 6EQT | Dock and adjust; build missing segments de novo | 16-42; 50-80; 84-167; 195-336; 374-454 | 162-167; 191-196; 284-317 |
|  | Iml3 | 245 | B | PDB 4JE3 | Dock and adjust | -1-243 |  |
| Ctf3 | Ctf3 | 733 | H |  | Build de novo | 43-66; 82-88; 104-120; 129-232; 297-397; 411-495 | 104-120; 149-end |
|  | Mcm16 | 181 | M |  | Build de novo | 70-128; 149-164 | All |
|  | Mcm22 | 239 | X |  | Build de novo | 71-129; 136-168; 175-208 | All |
| Cnn1 | Cnn1 | 361 |  |  | Dock; no refinement |  |  |
|  | Wip1 | 89 |  |  | Dock; no refinement |  |  |
| Unassigned | Ctf3 | n/a | Y |  | Build de novo | 653-678; 683-701; 1196-1213; 1667-1685 | All |
|  | Unknown | n/a | U |  | Build de novo | 1-23 | All |
