# Supplementary material for: The structure of the Ctf19c/CCAN from budding yeast
Source: eLife. 2019 Feb 14;8:e44239. doi: 10.7554/eLife.44239 (PMC6407923; doi:10.7554/eLife.44239)
Supplement: Supplementary file 3. — Genotypes and names of yeast strains used in this study. [file elife-44239-supp3.docx]

**Supplementary file 3 – Yeast strains used in this study (all strains built in the S288C background)**

| **Strain Number** | **Genotype** | **Reference** |
| --- | --- | --- |
| S288C (BY4741) | *MAT*a *his3*Δ*1 leu2*Δ*0 LYS2 met15*Δ*0 ura3*Δ*0* |  |
| SMH362 | *MATa CTF3-GFP::HisMX MTW1-mCherry::NatMX* | This study |
| SMH576 | *MATa CTF3-GFP::HisMX MTW1-mCherry::NatMX MCM21-3FLAG::KanMX* | This study |
| SMH591 | *MATa CTF3-GFP::HisMX MTW1-mCherry::NatMX ctf19Δ::KanMX* | This study |
| SMH580 | *MATa CTF3-GFP::HisMX MTW1-mCherry::NatMX mcm21-Δ95-3FLAG::KanMX* | This study |
| SMH530 | *MATa CTF19-GFP::HisMX MTW1-mCherry::NatMX* | This study |
| SMH531 | *MATa CTF19-GFP::HisMX MTW1-mCherry::NatMX ctf3Δ-KanMX* | This study |
| SMH595 | *MATa CTF19-GFP::HisMX MTW1-mCherry::NatMX MCM21-3FLAG::KanMX* | This study |
| SMH596 | *MATa CTF19-GFP::HisMX MTW1-mCherry::NatMX mcm21-Δ95-3FLAG::KanMX* | This study |
