# Supplementary material for: The structure of the Ctf19c/CCAN from budding yeast
Source: eLife. 2019 Feb 14;8:e44239. doi: 10.7554/eLife.44239 (PMC6407923; doi:10.7554/eLife.44239)
Supplement: Supplementary file 4. — Protein expression constructs for Ctf19c subunits are listed. [file elife-44239-supp4.docx]

**Supplementary file 4 – Protein expression constructs used in this study**

| **Plasmid** | **Coding Sequences** |
| --- | --- |
| pSMH104 | pLIC-Tra His6-TEV-Chl4; His6-TEV-Iml3 (Hinshaw and Harrison, 2013) |
| pSMH145 | pLIC-Tra His6-TEV-Ctf3; His6-TEV-Mcm16; His6-TEV-Mcm22 *(Hinshaw et al., 2017)* |
| pSMH1193 | pLICBac Cnn1 (untagged) |
| pSMH1198 | pLICBac His6-Wip1 |
| pFS1 | pET3aTra His6-TEV-Mcm21; Ctf19 (untagged) |
| pSMH1386 | pLIC-Tra Ctf19 (untagged); His6-TEV-Mcm21-Δ95 |
| pFS174 | pLIC-Tra His6-TEV-Nkp1; Nkp2 (untagged) *(Schmitzberger et al., 2017)* |
| pTDAO | pET28 Ame1-6His; Okp1 (untagged) |
| pSMH1164 | pLIC-Tra Cse4^1-50^-TEV-MBP-His6 |
| pSMH1189 | pLIC-Tra Cse4^1-50^-S22D, S33D, S40D-TEV-MBP-His6 |
